# Integrating filter paper extraction, isothermal amplification, and lateral flow dipstick methods to detect *Streptococcus agalactiae* in milk within 15 min

**DOI:** 10.3389/fvets.2023.1100246

**Published:** 2023-02-16

**Authors:** Lingling Zhu, Fengju Gong, Xia Liu, Xueqiang Sun, Yong Yu, Jie Shu, Zihao Pan

**Affiliations:** ^1^OIE Reference Laboratory for Swine Streptococcosis, College of Veterinary Medicine, Nanjing Agricultural University, Nanjing, China; ^2^College of Veterinary Medicine, Nanjing Agricultural University, Nanjing, China; ^3^Department of Detection and Diagnosis, Guizhou Animal Disease Prevention and Control Center, Guizhou, China; ^4^Department of Animal Diseases, China Animal Health and Epidemiology Center, Qingdao, China

**Keywords:** *Streptococcus agalactiae*, filter paper extraction, isothermal amplification, lateral flow dipsticks, rapid detection

## Abstract

**Introduction:**

Mastitis is one of the most serious diseases affecting dairy farming, causing huge economic losses worldwide. *Streptococcus agalactiae* is the main pathogenic bacterium of contagious mastitis and can deliver a devastating blow to a farm's economy. Rapid detection is the key to disease control.

**Methods:**

In this study, a rapid detection method for *S. agalactiae* was established. This method combines filter paper extraction, multienzyme isothermal rapid amplification (MIRA), and lateral flow dipsticks (LFD). To simplify the extraction procedure, we designed a disposable extraction device (DED). First, DED performance was evaluated by polymerase chain reaction (PCR) and then the lysis formula and extraction time were optimized. Second, this study compared the extraction performance of a filter paper and an automatic nucleic acid extraction instrument. After screening primers, MIRA for *S. agalactiae* was established and combined with LFD. Specificity and sensitivity were evaluated after optimizing the reaction conditions.

**Results:**

The results showed that the lowest extraction line for DED was 0.01–0.001 ng/μl. In the specificity study, 12 different bacteria were tested, and only *S. agalactiae* was found to be positive. In the sensitivity study, seven dilution gradients were established, and the lowest detection line was 3.52 × 10^2^ CFU/ml.

**Discussion:**

In summary, the method established in this study does not require laboratory equipment and is suitable for on-site detection. The entire method takes only 15 min, is low in cost, has high precision and low technical requirements for operators, which is in contrast with the high cost and cumbersome operation of traditional methods, and is suitable for on-site testing in areas with limited facilities.

## 1. Introduction

Mastitis is a devastating disease that causes severe economic losses to the dairy industry. It is defined as inflammation of mammary tissues caused by the invasion of various pathogens, including *Enterobacteriaceae, Staphylococcus*, and *Streptococcus* (1–3). *Streptococcus agalactiae*, also known as group B streptococcus, is a primary contagious pathogen and is commonly responsible for chronic subclinical mastitis in dairy cows because it forms a bacterial biofilm in the mammary tissue and stays in the mammary gland (4), leading to a decreased quality of dairy products. Moreover, *S. agalactiae* is also known to infect humans and aquatic animals, causing bacterial meningitis in newborns, puerperal septicemia during pregnancy, and massive mortality in aquatic animals (5). Although the serotypes and sequence type (ST) of *S. agalactiae* in bovines, humans, and fish are different, there is slight overlap, and some studies suggest that *S. agalactiae* is likely a zoonotic pathogen, making it of great public health relevance (6). Measures against the spread of *S. agalactiae* have been taken in most countries since the 20th century (7), but in China, *S. agalactiae* remains a major threat to the dairy industry (3, 8).

Timely diagnosis is essential to prevent the spread of contagious diseases. At present, polymerase chain reaction (PCR) is predominantly used to diagnose mastitis pathogens. However, this method is time-consuming and provides an opportunity for pathogen transmission. Recently, isothermal amplification has been developed, which allows nucleic acid amplification at constant temperatures and does not require thermal cycling. A study in 2000 published a method for isothermal amplification called loop-mediated isothermal amplification (LAMP), which uses four primers and deoxyribonucleic acid (DNA) polymerase to amplify double-stranded DNA (9). Nevertheless, this method requires more expensive enzymes and complex primers, resulting in a disadvantage in terms of cost and primer design procedure (10). Helicase-dependent isothermal DNA amplification (HDA) is a method that mimics *in vivo* DNA amplification (11). Instead of cycling temperatures, a specific helicase is used to open the DNA double strand, leading to isothermal amplification. However, HDA is still time-consuming, which limits its application in the field. Thus, a novel isothermal amplification method, multi-enzyme isothermal rapid amplification (MIRA), which is a modified version of recombinase polymerase amplification (RPA), has been developed. Compared with RPA, MIRA has the advantages of lower cost and shorter time. It is an ideal alternative to LAMP as only a pair of primers can provide exponential amplification at constant temperature. When MIRA is combined with lateral flow dipsticks (LFD), a direct visual readout is produced, eliminating reliance on laboratory equipment.

Although MIRA in combination with LFD is convenient, extraction and purification of nucleic acids remain a major problem in the detection procedure. Traditional liquid-phase methods generally extract nucleic acids by separating and denaturing proteins, polysaccharides, and other components. These procedures require extensive pipetting and centrifugation steps, which are time-consuming and labor-intensive (12). This has led to the rise of solid-phase extraction methods, such as extraction with silica and magnetic beads, which purify nucleic acids by binding DNA to the solid phase *via* salt bridges at high salt concentrations and breaking the bridges at low salt concentrations (13–15). Although this method considerably reduces extraction time, it still cannot be performed outside the laboratory. A study published in 2017 found that cellulose filter paper, commonly used in the laboratory, could capture nucleic acid molecules in liquids and could be used in amplification reactions (16). This research confirmed that nucleic acids extracted from cellulose filter paper could be retained in multiple washing steps and that a desired quantity of amplicons could be created for subsequent amplification reactions. Additionally, the economical features of the filter paper mean that it is an ideal novel medium for nucleic acid adsorption.

In this study, we developed a filter paper-based disposable nucleic acid extraction device and combined it with MIRA–LFD, which is also a convenient and rapid method. The objective of this study was to provide a rapid, convenient, laboratory-free method for the clinical diagnosis of *S. agalactiae*, particularly for use in resource-limited areas.

## 2. Materials and methods

### 2.1. Strains, culture conditions, and DNA extraction

Reference strains of *S. agalactiae, Streptococcus uberis, Streptococcus dysgalactiae, Staphylococcus aureus, Escherichia coli, Klebsiella pneumoniae, Pasteurella multocida, Salmonella, Proteus mirabilis, Bacillus cereus, Serratia marcescens*, and *Shigella sonnei* were kept in our laboratory. All strains were grown at 37°C at 150 revolutions per minute (rpm) for 12 h. Genomic DNA was extracted with a D3350 bacterial DNA kit purchased from Omega.

### 2.2. Design of primers and probes

The primers and probe used in this study are listed in Table 1 and were designed using the program “Primer premier5.” All primers were synthesized by Sangon Biotech Co. MIRA primers were designed with the *S. agalactiae cfb* gene (GeneBank accession number: NZ_LR134512.1). The reserve primers of MIRA–LFD were labeled with biotin at the 5' end. The probes were also designed with the *S. agalactiae cfb* gene (GeneBank accession number: NZ_LR134512.1) using the program “Primer premier5” and were labeled with carboxyfluorescein (FAM) at the 5' end and a C3-spacer at the 3' end. The tetrahydrofuran (THF) site was 31 bp at the 5' end and 17 bp at the 3' end.

**Table 1:** Primers and probes used in this experiment.

**Method**	**Primer name**	**Sequence (5^′^-3^′^)**
PCR	S.d-F	GAACACGTTAGGGTCGTC
	S.d-R	AGTATATCTTAACTAGAAAAACTATTG
	*S. aureus*-F	TCTTCAGAAGATGCGGAATA
	*S. aureus*-R	TAAGTCAAACGTTAACATACG
	*E. coli*-F	GTAACGTTTCTACCGCAGAGTTG
	*E. coli*-R	AGGGTTGGTACACTGTCATTACG
	S.a-F	CCCTCTCAGGTCGGCTATGTATCGTCGCCTTGGTG
	S.a-R	GACAAAAAAACAATAAATCTGTCAGCGAGACAGAA
MIRA	Cfb-F	ACTGAAGCAAATGGATCTAAAATGCGAATAAC
	Cfb-R1	TTGAGAAATATCAAAGATAATGTTCAGGGA
	Cfb-R2	GAACTCTAGTGGCTGGTGCATTGTTATTTT
	Cfb-R3	TAATAATCAAGCCCAGCAAATGGCTCAAAAGC
	Cfb-R4	ACAACTCCACAAGTGGTAAATCATGTAAATAGTA
	Cfb-R5	GTGCATTGTTATTTTCACCAGCTGTATTAGAAGTA
	Cfb-R6	GATTCAATTAAAGCTCAAGTTAACGATGTA
	Cfb-R7	AACTCCACAAGTGGTAAATCATGTAAATAG
MIRA–LFD	Cfb-F	ACTGAAGCAAATGGATCTAAAATGCGAATAAC
	Cfb-R1-bio	Biotin-TTGAGAAATATCAAAGATAATGTTCAGGGA
	Cfb-R2-bio	Biotin-GAACTCTAGTGGCTGGTGCATTGTTATTTT
	Cfb-R3-bio	Biotin-TAATAATCAAGCCCAGCAAATGGCTCAAAAGC
	Cfb-R5-bio	Biotin-GTGCATTGTTATTTTCACCAGCTGTATTAGAAGTA
	Cfb-R7-bio	Biotin-AACTCCACAAGTGGTAAATCATGTAAATAG
	Cfb-P	FAM-AAGTCTTTAATTTTTCAACGCTAGTAATAGC(THF)TCATTAACCGGTTTTTC-C3

### 2.3. PCR

The polymerase chain reaction was performed in a 20-μl mixture system consisting of 10 μl of Green Taq Mix (Vazyme), 6 μl of sterile distilled water, 1 μl of forward (F) and reverse (R) primers (10 μM), and 2 μl of the template (DNA solution or a filter paper in this study). Reaction conditions included an initial pre-denaturation at 95°C for 5 min, followed by 35 cycles at 95°C for 30 s, annealing at 55°C for 30 s, elongation at 72°C for 1 min 30 s, and a final extension at 72°C for 5 min. Amplicons were detected using 1% agarose gel electrophoresis (AGE).

### 2.4. DNA extraction capability assessment of a filter paper

Aliquots of 20 μl of 10-fold serially diluted DNA (ranging from 100 to 0.001 ng/μl) of *S. aureus, E. coli, S. dysgalactiae*, and *S. agalactiae*, respectively, were added to filter paper discs (Whatman No. 6, Whatman) with a diameter of 3 mm. After 10 s, the filter paper was removed and placed into PCR as a template. Pure DNA was used as the positive control.

### 2.5. Preparation and construction of a disposable extraction device

**Figure 1:**
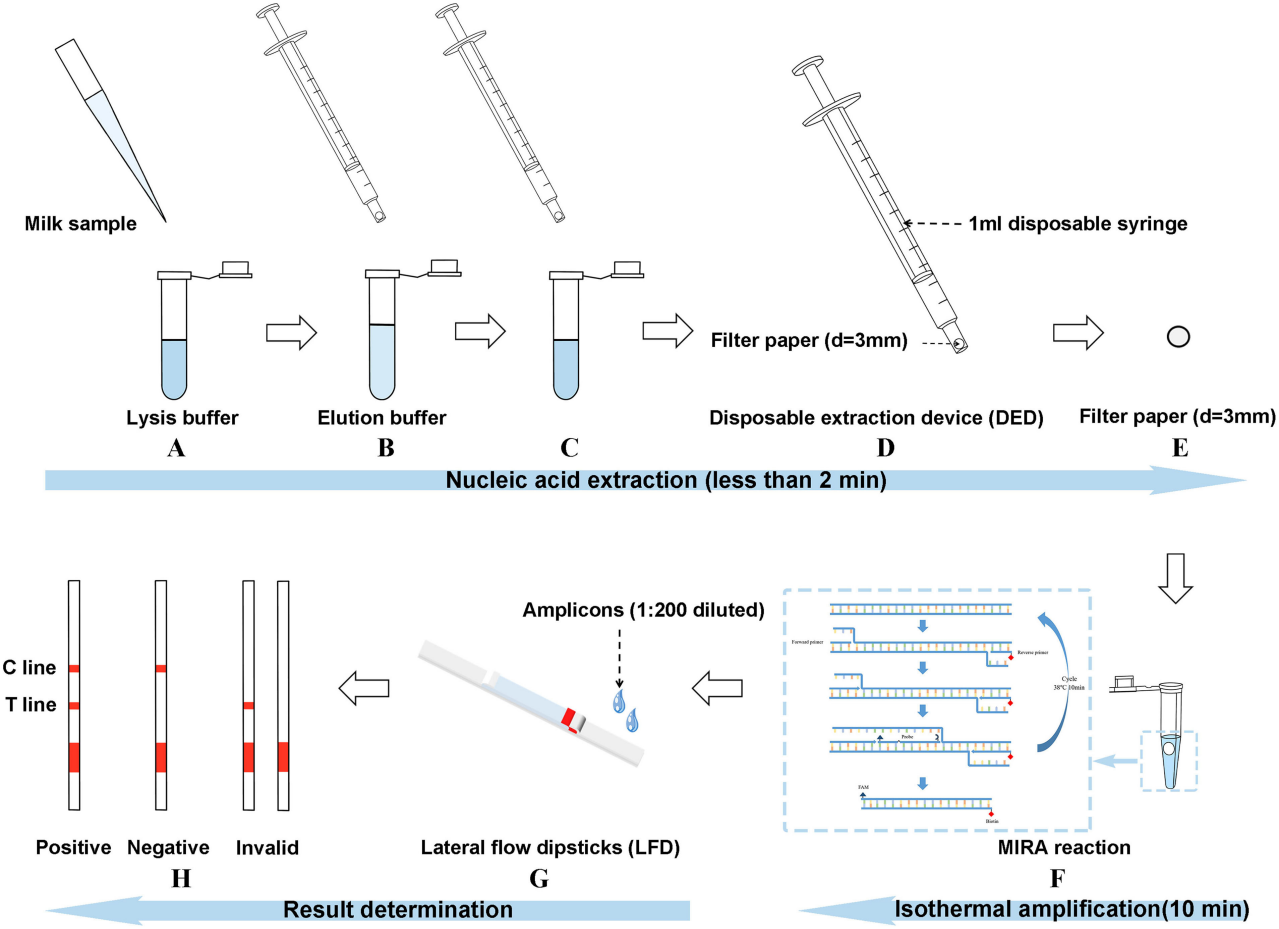
Introduction of the three-stage method [multienzyme isothermal rapid amplification–lateral flow dipstick–disposable extraction device (DED–MIRA–LFD)]. **(A)** The milk sample was added to the lysis buffer for lysing. **(B)** DED was used to blow the mixture up and down for 30 s. **(C)** DED was used to aspirate the elution buffer for 20 times. **(D)** The syringe was emptied. **(E)** The filter paper was taken out. **(F)** The filter paper into the MIRA reaction was used as a template. **(G)** Amplicons were detected by LFDs after the step of 1:200 dilution. **(H)** Judgment of result.

The disposable extraction device (DED) consisted of a 1-ml needleless injector and a filter paper disc (Figure 1). The filter paper disc (diameter of 3 mm) was placed on the top of a needleless injector without any modification. DED preparation was completed in a sterile environment and subsequently exposed to ultraviolet (UV) radiation for 24 h. Firstly, 100 μl of milk samples were added to 500 μl of lysis buffer (1.5 M guanidine isothiocyanate, 50 mM Tris-HCl [pH 8.0], 20 mM EDTA, 1% Tween-20, 2 mg/ml DTT, 40 mg/ml Triton 100, a certain concentration of NaCl) in a 1.5-ml centrifuge tube; after shaking and mixing, the mixture was sucked and blown for a certain time (determined in subsequent experiments) with the DED. In this step, bacterial genomic DNA was released and captured using a filter paper. The subsequent washing step was performed by sucking up and down for a certain time (determined in subsequent experiments) in 200 μl of washing buffer (10 mM Tris-HCl [pH 8.0], 0.1% Tween-20) to remove amplification inhibitors. Finally, the filter paper was used as a template for subsequent nucleic acid amplification assay.

### 2.6. Screening of the optimal NaCl concentration during lysis

The concentration of NaCl in the lysis buffer influenced the extraction effect. To determine the optimal NaCl concentration, we set up 0, 50, 100, 150, and 200 mM NaCl concentration gradients separately. Lysis time was 1 min; 10 washes took place. After extraction, the filter paper was used as a template for PCR.

### 2.7. Screening of the optimal lysis time and elution number

The lysis time and elution number also impacted the extraction effect. To identify the optimal lysis time and elution number, firstly, we set different lysis times (30 s, 1 min, 1 min 30 s, 2 min, 2 min 30 s, and 3 min) combined with 10 times of elution for nucleic acid extraction using the DED. The results were analyzed by PCR; the reaction time that generated a strong band was selected for further experiments. Subsequently, we set various elution numbers (3, 5, 10, 15, and 20 times) combined with the optimal lysis time for nucleic acid extraction using the DED. The results were analyzed by PCR; the time that created the brightest band was selected for subsequent testing. Each assay was performed in triplicate.

### 2.8. Comparison of nucleic acid extraction ability between the DED and magnetic bead method

The DED procedure was conducted using the optimal lysis time and elution number. The magnetic bead method was performed with a commercial magnetic bead extractor (Vazyme). The results were compared by PCR.

### 2.9. MIRA reaction

The MIRA-basic reaction (amplification future) was performed in a 25-μl mixture consisting of 14.7 μl of buffer A (supplied in the kit), 5 μl of sterile distilled water, 1 μl of forward (F) and reverse (R) primers (10 μM), 2 μl of a template (DNA solution or a filter paper in this study), and 1.3 μl of buffer B (supplied in the kit); subsequently, the reaction was incubated at 38°C for 30 min. The amplicons were purified using DNA extraction reagent 25:24:1 (ACMEC) at a ratio of 1:1 and then detected using 1% AGE.

The MIRA–LFD reaction (amplification future) was conducted in a volume of 25 μl containing 14.7 μl of buffer A (supplied in the kit), 4.75 μl of sterile distilled water, 1 μl of forward (F) and reverse (R) primers (10 μM), 0.3 μl of probe (10 μM), 2 μl of the template (DNA solution or a filter paper in this study), and 1.25 μl of buffer B (supplied in the kit); then, the reaction was incubated at the given temperature for a specific time. Amplicons were detected using LFDs (Gu'An Beiji) after a step of 1:200 dilution.

### 2.10. Primer screening for MIRA–LFD

Primer screening for MIRA–LFD was first performed with Cfb-F, Cfb-R1, Cfb-R2, Cfb-R3, Cfb-R4, Cfb-R5, Cfb-R6, and Cfb-R7 using the MIRA-basic reaction; primers that generated a strong and single band were selected for further testing. Afterward, to prevent false positive results caused by reverse primer and probe mismatches, we set up two reaction conditions: negative and positive reactions. The negative was in accordance with the MIRA–LFD reaction but used pure water as a template. The positive reaction was the normal MIRA–LFD reaction. The primer, which emerged with one band in the negative reaction and two bands in the positive reaction, was chosen for subsequent experiments.

### 2.11. Optimization of reaction conditions for MIRA–LFD

To determine the best reaction conditions for MIRA–LFD, we set different temperature gradients (25, 30, 35, 37, 38, 39, 40, and 45°C) and reaction times (5, 10, 15, 20, 25, and 30 min). The temperatures and reaction times, which generated strong bands, were chosen for further testing.

### 2.12. Specificity and sensitivity of the complete method

The complete method (DED–MIRA–LFD) consisted of a DED for extraction and MIRA–LFD for amplification and detection. To determine the specificity and sensitivity of DED–MIRA–LFD with the selected primer, 10 μl of the bacterial solution was added to 90 μl of sterile milk as artificially spiked milk. Specificity was conducted with 12 species of bacteria-spiked milk including *S. agalactiae*; the negative control included a sterile milk sample instead of a spiked milk sample. Sensitivity was validated using a 10-fold serially diluted bacterial solution of spiked milk ranging from 10^1^ to 10^7^ CFU/ml.

### 2.13. Detection of clinical samples

In 2021, a total of 48 clinical milk samples were collected from a dairy farm in Liaoning, Guangdong, Heilongjiang, Shandong, and Ningxia autonomous regions of China. The samples were stored in our laboratory and were submitted by ranchers. DED–MIRA–LFD was performed to compare the coincidence rate using bacterial isolation as a gold standard.

### 2.14. Statistical analysis

Kappa (κ) was used to compare the results of DED–MIRA–LFD and the bacterial isolation assay. Statistical analysis was performed with the Statistical Package for the Social Sciences (SPSS; version 26.0).

## 3. Results

### 3.1. DNA extraction capability assessment of a filter paper

**Figure 2:**
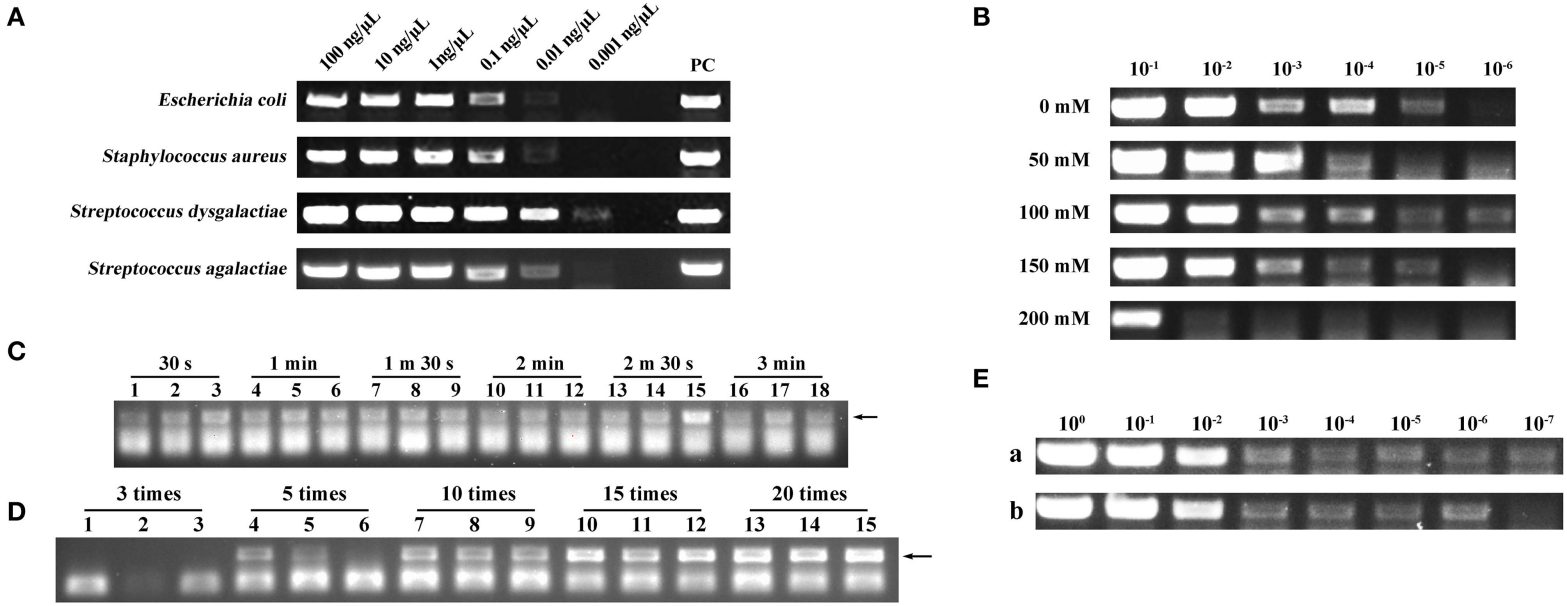
**(A)** Deoxyribonucleic acid (DNA) extraction capability assessment of a filter paper. PC represents positive control. **(B)** Screening of optimal NaCl concentration in lysis. 10^−1^, 10^−2^, 10^−3^, 10^−4^, 10^−5^, and 10^−6^ represent the bacterial dilution gradient. Concentrations of NaCl were 0, 50, 100, 150, and 200 mM. **(C)** Screening of optimal lysis number (30 s, 1 min, 1 min 30 s, 2 min, 2 min 30 s, and 3 min). Each assay was conducted in triplicate. **(D)** Screening of optimal elution number (3, 5, 10, 15, and 20 times). Each assay was conducted in triplicate. **(E)** Comparison of nucleic acid extraction ability between the disposable extraction devices (DEDs) and magnetic bead method. (a) Magnetic bead extraction method. (b) Filter paper extraction method. 10^0^, 10^−1^, 10^−2^, 10^−3^, 10^−4^, 10^−5^, 10^−6^, and 10^−7^ represent the bacterial dilution gradient.

To investigate whether a filter paper could extract nucleic acids, we used a filter paper to extract DNA from four different species of microbes. As shown in Figure 2A, the filter paper extraction method had the ability to extract DNA and the lowest extraction limit was 0.01–0.001 ng/μl. Conventional nucleic acid extraction methods are complex and cannot be operated without laboratory conditions (17). In this study, we designed a new DED using a filter paper extraction method, and nucleic acids could be captured using a filter paper only by sucking and blowing the lysis and elution buffer. A simple manipulation shortened the extraction time to <2 min.

### 3.2. Screening of the optimal NaCl concentration during lysis

The concentration of NaCl in the lysis buffer can impact the extraction performance of the DED. Here, we set up NaCl concentration gradients of 0, 50, 100, 150, and 200 mM separately; the NaCl concentration of 100 mM exhibited higher extraction efficiency (Figure 2B).

### 3.3. Screening of the optimal lysis time and elution number

The performance of the DED is also dependent on the lysis time and elution number. Therefore, we screened different lysis times (30 s, 1 min, 1 min 30 s, 2 min, 2 min 30 s, and 3 min) and elution numbers (3, 5, 10, 15, and 20 times). As shown in Figure 2C, different lysis times did not have a distinct impact on DNA extraction; therefore, we chose the shortest lysis time for our method. For elution times, to obtain ideal sensitivity, 20 times of elution was selected as the best elution time due to the high production in the PCR assay (Figure 2D). Consequently, 30 s of lysis time and 20 times of elution were chosen as the best combination in this study.

### 3.4. Comparison of nucleic acid extraction ability between the DED and the magnetic bead method

Once the DED was in place, we compared its performance against the magnetic bead method. Although the DED was not as efficient as the commercial magnetic bead method (Figure 2E), it had a more favorable extraction time (<2 min) and lower cost.

### 3.5. Screening of a primer for MIRA–LFD

**Figure 3:**
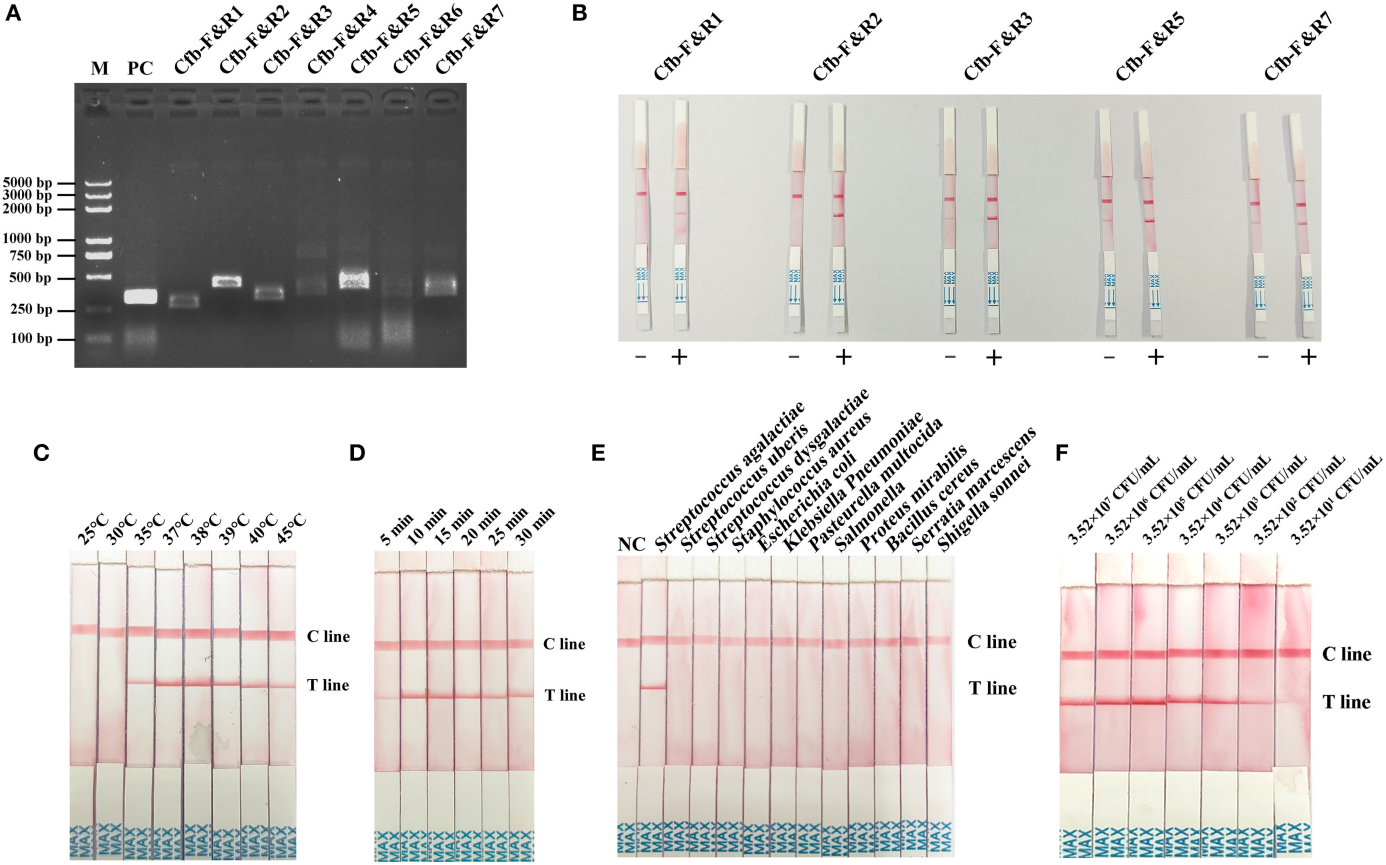
**(A)** Primer primary screening by the MIRA reaction. M = Marker, PC = positive control, Cfb-F&R1, Cfb-F&R2, Cfb-F&R3, Cfb-F&R4, Cfb-F&R5, Cfb-F&R6, and Cfb-F&R7 = primer pairs. **(B)** Primer rescreening by MIRA–LFD reaction. “–” = a negative reaction which utilized pure water as a template. “+” = a positive reaction followed by the normal MIRA–LFD reaction. Cfb-F&R1, Cfb-F&R2, Cfb-F&R3, Cfb-F&R5, and Cfb-F&R7 = primer pairs. **(C)** Reaction temperature screening for MIRA–LFD. C line = the control line, T line = the test line. **(D)** Reaction time shifting for MIRA–LFD. C line = the control line, T line = the test line. **(E)** Specificity of the complete method (DED–MIRA–LFD). NC = negative control. C line = the control line, T line = the test line. **(F)** Sensitivity of the complete method (DED–MIRA–LFD).

Primer screening for MIRA–LFD was first performed using the MIRA-basic reaction. Primers Cfb-F&R1, Cfb-F&R2, Cfb-F&R3, Cfb-F&R5, and Cfb-F&R7 produced a single strong band at 1% AGE (Figure 3A). Subsequently, we discarded the primer pairs that produced false positive results in the MIRA–LFD reaction and selected strong bands for subsequent testing. Finally, the Cfb-F&R2 primer was used for subsequent studies (Figure 3B).

### 3.6. Optimization of reaction conditions for MIRA–LFD

To identify the best reaction condition, we set different temperature gradients (25, 30, 35, 37, 38, 39, 40, and 45°C) and reaction times (5, 10, 15, 20, 25, and 30 min). In the screening of temperatures, the intensity of the bands became clearer upon reaching 38°C and gradually decreased above 38°C (Figure 3C). With regard to time, 10 min revealed the strongest band (Figure 3D). Consequently, 38°C for 10 min was chosen as the optimal reaction condition.

### 3.7. Specificity and sensitivity of the three-stage method (DED–MIRA–LFD)

To determine specificity, the genomic DNA of 12 bacterial species in artificially spiked milk was extracted using DED for the MIRA–LFD reaction. Only *S. agalactiae* was successfully detected; no other bacterial species were detected (Figure 3E). The results confirmed that the method established here was highly specific for *S. agalactiae*. The analytical sensitivity of DED–MIRA–LFD was determined using a 10-fold serially diluted bacterial solution in spiked milk ranging from 10^1^ to 10^7^ CFU/ml. The results were observed when the bacterial concentration reached 3.52 × 10^2^ CFU/ml (Figure 3F).

### 3.8. Detection of clinical samples

To evaluate the performance of DED–MIRA–LFD, 48 clinical milk samples were detected *via* bacterial isolation and DED–MIRA–LFD. A total of 35 samples were detected as positive using DED–MIRA–LFD, and 36 (75%) were detected as positive using bacterial isolation. The bacterial isolation method detected 12 negative samples, while the DED-MIRA-LFD detected 13 negative samples (Table 2). The results of DED–MIRA–LFD were in good agreement with those of bacterial isolation (κ = 0.946).

**Table 2:** Comparison of bacterial isolation and DED–multienzyme isothermal rapid amplification (MIRA)–lateral flow dipstick (LFD) results for clinical samples.

		**DED-MIRA-LFD**			**Kappa (κ)**
		**Positive**	**Negative**	**Total**	
Bacteria isolation	Positive	35	1	36	0.946
	Negative	0	12	12	
	Total	35	13	48

## 4. Discussion

The development of point-of-care testing, especially during the outbreak of COVID-19, is currently proceeding at an unprecedented rate. Molecular techniques are considered an important method of pathogen diagnosis that shares the advantages of high sensitivity and specificity, rapidity, and automation (18). Specific and rapid diagnostic methods are beneficial to the prevention and control of infectious diseases. *S. agalactiae* is the main infectious pathogen of mastitis, leading to a significant increase in the somatic cell count in milk, which is an important indicator of milk quality (19). Here, we established a more convenient, rapid, and economical detection method, which combined filter paper extraction, isothermal amplification, and LFD, shortening the detection time to 15 min. Furthermore, the method can directly extract microbial nucleic acids from milk, is convenient and rapid, and has a low false positive rate, compared with the high cost and cumbersome laboratory detection methodology of *S. agalactiae*.

Nucleic acid extraction is crucial for both PCR and isothermal amplification. Filter paper extraction is a rapid method developed in recent years, but extraction efficiency has been a limiting factor for the application of this method (16). It is believed that the smaller the pore size of the filter paper, the greater the number of nucleic acids that can be adsorbed or bound. In the current study, a smaller pore-size filter paper was selected, resulting in an increase in the minimum extraction limit (0.01–0.001 ng/μl) compared to a previous study (0.1 ng/μl) (20). Screening of the concentration of NaCl showed that 100 mM NaCl concentration had better extraction ability, which was similar to that screened by Liu et al. (21). In this result, we observed that the extraction effect continued to improve as the concentration of NaCl increased from 0 to 100 mM but tended to decrease when the concentration of NaCl increased from 100 to 200 mM. This may be due to the fact that, at a concentration of 0–100 mM, the elevation of the concentration of NaCl neutralizes the negative charge of cellulose filter paper and DNA, leading to the weakening of the repellent electrostatic forces. However, as the concentration of NaCl increased, the amount of DNA binding to a filter paper increased, but the high concentration of salt remaining on the filter paper inhibited the amplification reaction, thus reducing the amplification yield. The filter paper extraction method established in our study was not as sensitive as the magnetic bead extraction method, and this may be because the adsorption surface area of the filter paper is not as large as that of magnetic beads. Additionally, due to the capillarity of a filter paper, it can absorb more amplification inhibitors, which are difficult to wash out. The price per sample of DEDs described here is $US 0.11 including a 1-ml injector and reagents (Table 3). These devices can be used not only for the extraction of *S. agalactiae* but also for the extraction of nucleic acids from all pathogenic microbes in milk samples, which has a promising application in the field of rapid diagnostics.

**Table 3:** The time required and cost for the disposable extraction device (DED) and the magnetic bead method.

	**Disposable extraction device (DED)**	**Magnetic bead method**
Time required	<2 min	15 min
Cost per sample (USD)	$0.11	$5.8

In terms of DNA amplification, traditional PCR and various isothermal amplification methods are dependent on laboratory instruments. Therefore, many studies have developed visualization assays aimed at simplifying the pathogen detection process in various fields (22–24). A combination of filter paper extraction, LAMP, and LFD can be used to detect various complex samples with a sensitivity of 10^1^ CFU/ml (25), which is lower than that of our study (10^2^ CFU/ml). However, our method shortened the detection time (15 min) by 75%, which could be crucial in the prevention and control of infectious diseases. Simultaneously, Tang et al. established an integrated method of nucleic acid extraction, amplification, and detection, which had lower sensitivity (10^3^ CFU/ml) and detection time (1 h) than our method (26). A 2019 study established an assay for the direct detection of *B. cereu* in milk, which was also performed by combining filter paper extraction with isothermal amplification, but the process was time-consuming, and the interpretation of the results was error-prone (21). In our study, sensitive primer pairs and probes were selected by extensive primer screening. The filter paper extraction method, isothermal amplification, and LFD were integrated, whose detection limit was 10^2^ CFU/ml, creating a visual interpretation of the results and on-site diagnostics.

In conclusion, the detection method established in our project detected *S. agalactiae* directly from milk samples, and the required equipment was only small instruments such as vortex meters. However, the problem of nucleic acid contamination becomes very serious due to the high sensitivity of MIRA. This contamination comes from aerosol pollution during nucleic acid extraction or is caused by opening the lid after amplification. Therefore, it is necessary to develop a completely sealed detection device to avoid DNA contamination. Moreover, by designing mobile detection stations or integrated detection devices, our method can be used for on-site diagnosis in areas with limited resources, such as dairy farms without a laboratory environment. This will be an important step forward in the prevention and control of mastitis on farms.

## Data availability statement

The original contributions presented in the study are included in the article/supplementary material, further inquiries can be directed to the corresponding author.

## Author contributions

Methodology: LZ. Writing—original draft preparation: LZ and JS. Writing—review and editing: ZP. Supervision: XL and YY. Project administration: FG and XS. All authors have read and agreed to the submitted version.
